# Apelin protects against myocardial ischemic injury by inhibiting dynamin-related protein 1

**DOI:** 10.18632/oncotarget.21777

**Published:** 2017-10-10

**Authors:** Wei Xu, Hongwei Yu, Ruixue Ma, Lina Ma, Qiushuang Liu, Huitong Shan, Chengyu Wu, Rong Zhang, Yuhong Zhou, Hongli Shan

**Affiliations:** ^1^ Department of Pharmacology (The State-Province Key Laboratories of Biomedicine Pharmaceutics of China, Key Laboratory of Cardiovascular Research, Ministry of Education), College of Pharmacy, Harbin Medical University, Harbin 150081, China; ^2^ Department of Cardiology, The Second Affiliated Hospital of Harbin Medical University, Harbin 150081, China; ^3^ Department of Histoembryology, Harbin Medical University, Harbin 150081, China

**Keywords:** apelin, cardiomyocyte apoptosis, mitochondrial fission, hypoxia, myocardial infarction

## Abstract

It is known that dynamin-related protein 1 (Drp1)-mediated mitochondrial fission plays an important role in ischemic injury of myocardial infarction (MI). Apelin, an endogenous ligand for Apelin receptor, acts as a key modulator of cardiovascular diseases. Here, we examined the effects of Apelin on MI injury and underlying mechanisms. Adult male C57BL/6J mice were treated with Apelin for 4 weeks and then subjected coronary artery ligation (LAD) to induce MI and the protective effects of Apelin on MI injury were evaluated at 6 h post LAD. Mitochondrial fission was significantly increased in MI as evidenced by enhanced expression of phosphorylated Drp1 (p-Drp1^ser 616^) without affecting total Drp-1 level and degenerative transformation of mitochondria into short rods as typical fission. Apelin markedly inhibited p-Drp1^ser 616^ and preserved mitochondrial morphology in MI. Similar effects of Apelin were consistently observed in primary cultured cardiomyocytes under hypoxia. Apelin decreased hypoxia-induced cardiomyocyte apoptosis as evidenced by decreased TUNEL-positive cells and preserved mitochondrial membrane potential (MMP). Apelin decreased Bax/Bcl-2 ratio and limited the release of cytochrome C and activation of caspase-9 and caspase-3 both *in vivo* and *in vitro*. Finally, Apelin diminished the infarct size and normalized the impaired cardiac function as indicated by rescuing of the decreased ejection faction and fractional shortening in MI mice. In conclusion, Apelin prevented mitochondrial fission by inhibiting p-Drp1^Ser616^, which prevents loss of MMP and inhibits the mitochondria-mediated apoptosis. These results indicate that the inhibition of Drp-1 activation by Apelin is a novel mechanism of cardioprotection against MI injury.

## INTRODUCTION

Myocardial infarction (MI) is still a primary cause of death in the world with an increased risk of morbidity and mortality [1–3]. Many cardioprotective pharmacological agents failed to exert their protective effects [4–6]. While many interventions are effective in reducing MI injury and offering limited protection against ischemia/reperfusion (I/R) injury [7, 8]. Therefore, exploring novel pharmacological agents to help salvage ischemic damages may benefit clinical outcomes for MI patients.

Mitochondria are a vital cell organelle in cardiomyocytes because of their pivotal role in ATP production required for myocardial contraction and survival. Mitochondria undergo cycles of fission (division) and fusion (joining), which play an important role in regulating apoptosis [9–11]. Increased mitochondrial fission in the ischemic heart contributes to apoptosis induction and infarct generation, while inhibiting mitochondrial fission reduces myocardial injury and improves cardiac function following MI [12, 13]. Dynamin-related protein-1 (Drp1) is a cytosolic protein that is recruited over the mitochondrial surface to initiate fission through interaction with binding partners as fission protein 1 (Fis1) [14]. Ding *et al.* [15] reported that Drp1-mediated mitochondrial fission is increased following I/R under diabetic conditions and its inhibition with Mdivi-1 reduces I/R injury and improves cardiac function. Grohm *et al.* found that Drp1 siRNA and small molecule inhibitors of Drp1 could prevent mitochondrial fission and loss of mitochondrial membrane potential [16]. Inhibition of Drp1 has been demonstrated to reduce mitochondrial fission and infarct size during ischemic injury in animals [17, 18]. Therefore, manipulating Drp1-dependent mitochondrial fission may be an alternative strategy for the development of new anti-apoptotic drugs.

Apelin is an endogenous ligand for the previously orphaned G-protein-coupled Apelin related receptor (APJ) that is involved in regulating cardiovascular functions. Apelin is known to have potent protective effect on heart and significant therapeutic potential for heart failure [19–23]. Rastaldo *et al.* found that Apelin can limit infarct size and improve cardiac post-ischemic mechanical recovery only if given after ischemia [24]. Tao *et al.* reported that Apelin protects the heart against I/R injury through inhibition of the ER (endoplasmic reticulum)-dependent apoptotic pathway [25]. Apelin inactivates GSK-3β resulting in inhibition of the opening of mitochondrial permeability transition pore by activating PI3K/Akt and ERK1/2 involving in the reperfusion injury salvage kinase (RISK) pathway. However, whether Apelin plays a functional role in mitochondrial dynamics in cardiomyocytes remains unknown. The aim of this study was to examine the effects of Apelin on MI injury and explored their underlying mechanisms based on Drp1.

## RESULTS

### Apelin inhibits mitochondrial fission in MI mice and hypoxic cardiomyocytes

**Figure 1:**
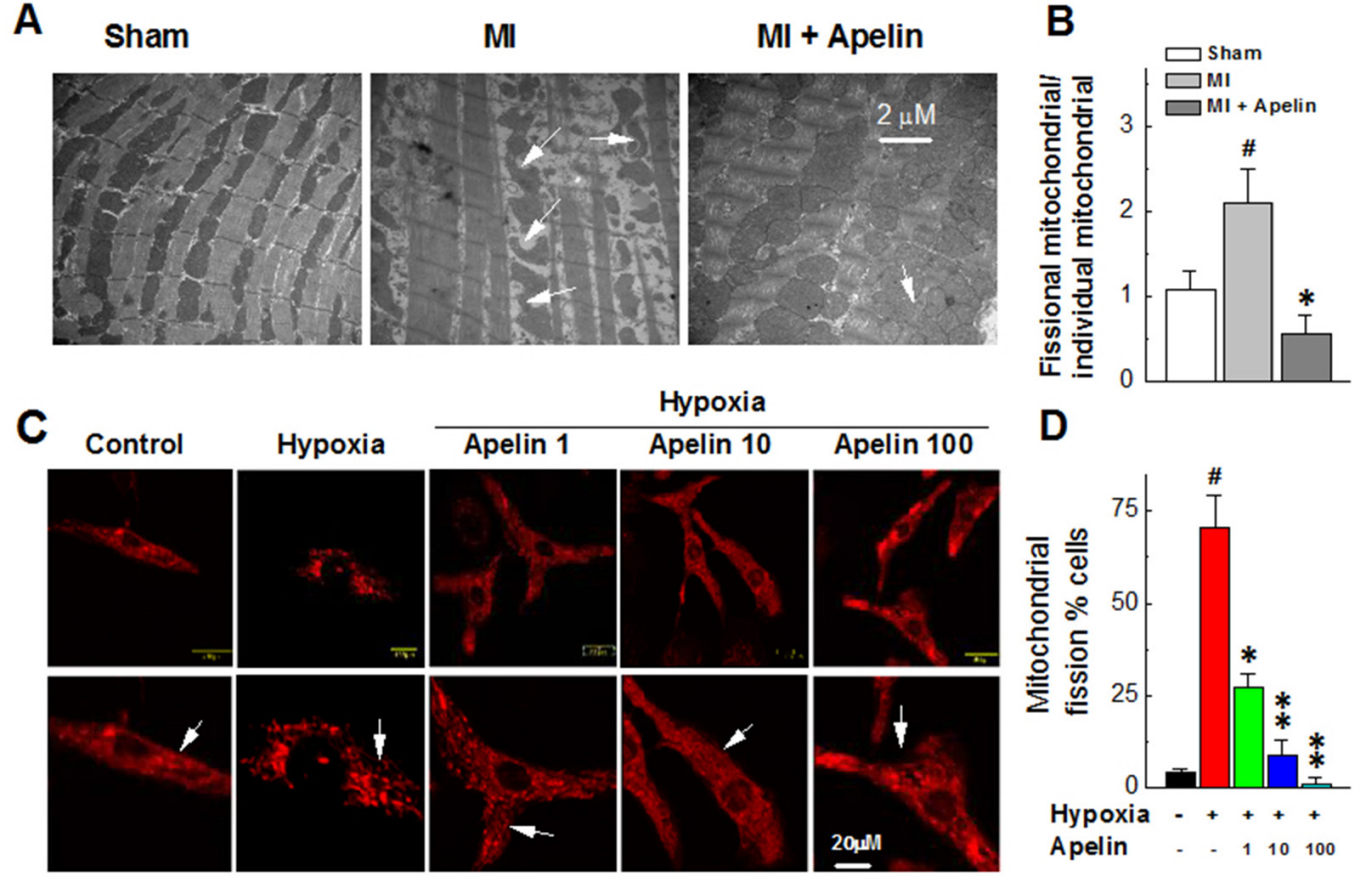
Apelin inhibits mitochondrial fission in MI mice and in hypoxic cardiomyocytes **(A)** Representative changes of mitochondrial morphology in MI mice under a transmission electron **(B)** Quantification of the ratio of fissional mitochondrial and individual mitochondrial. **(C)** Representative confocal microscopic images of mitochondrial morphology in cultured neonatal mice cardiomyocytes. **(D)** The representative electron microscopy images of mitochondrial morphology. Data are presented as mean ± SEM, ^#^*P* < 0.05 *vs* sham, *n* = 6; ^*^P < 0.05, ^**^P < 0.01 *vs* MI, *n* = 6. *P* values were analyzed using one-way ANOVA.

Pronounced changes of mitochondrial morphology were consistently observed in the areas proximal to infarct/ischemic zone in MI mice under a transmission electron microscope. Specifically, ischemia decreased mitochondrial size, indicating an enhanced mitochondrial fission. This damaging effect was attenuated in mice treated with Apelin (Figure 1A & 1B).

Similar alterations of mitochondria were consistently observed in cultured neonatal mice cardiomyocytes. As illustrated in Figure 1C & 1D, confocal images revealed that the mitochondria existed a long and filamentous or tubular-shaped structure in cardiomyocytes under a normoxic condition, whereas they degenerated into short, round-shaped morphology like small spheres or short rods typical of mitochondrial fission in cardiomyocytes under a hypoxic condition (hypoxia caused nearly an eightfold increase in the number of cells with mitochondrial fission). With Apelin treatment, the number of cardiomyocytes with mitochondrial fission induced by hypoxia was substantially decreased (Figure 1C & 1D).

### Apelin reduces the activity of Drp1

**Figure 2:**
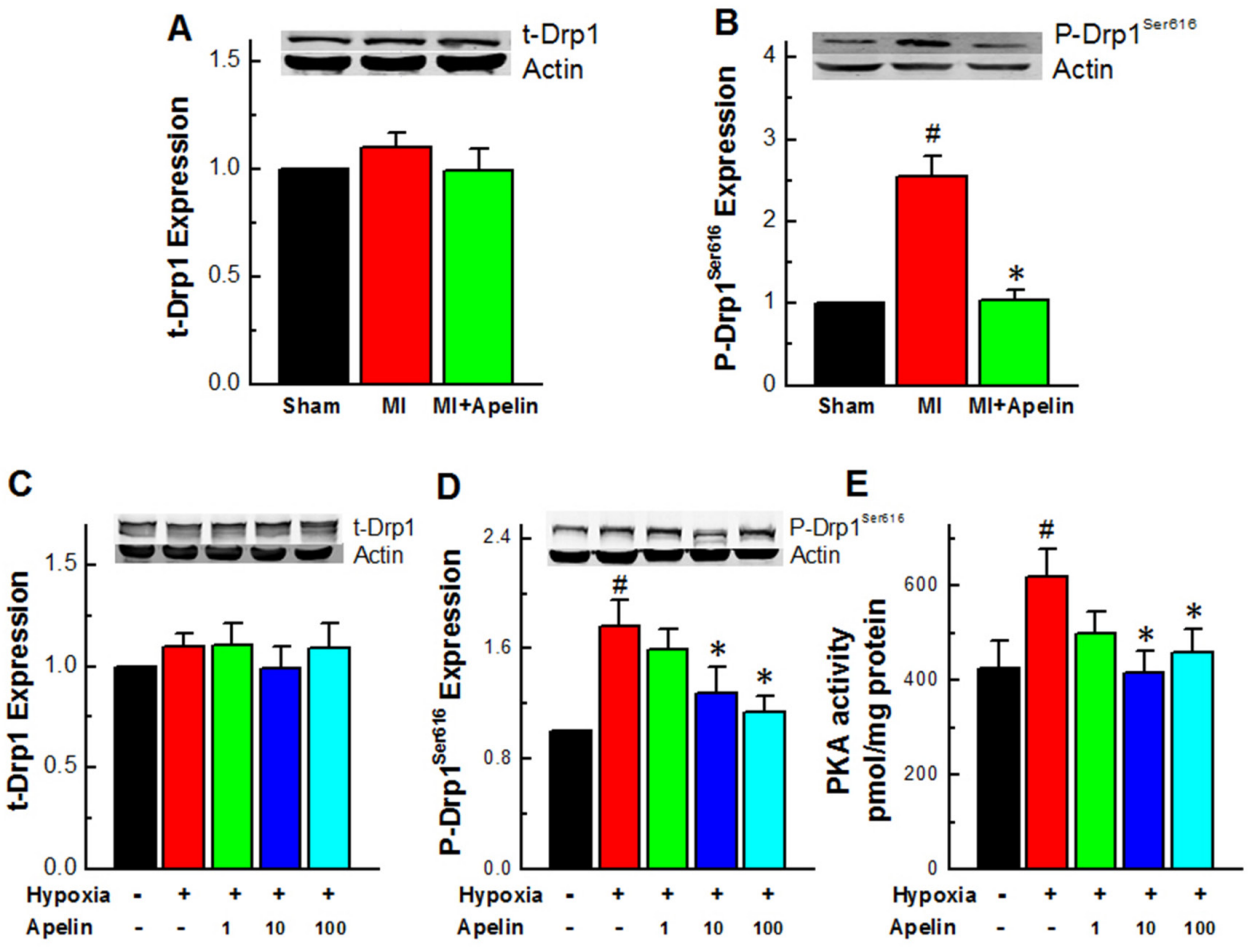
Apelin reduces the activity of Drp1 **(A)** Expression levels of Drp1 in MI mice. **(B)** Expression levels of p-Drp1^Ser616^ in MI mice. **(C)** Expression levels of Drp1 in cultured neonatal mice cardiomyocytes. **(D)** Representative western blot band of p-Drp1^Ser616^ in cultured neonatal mice cardiomyocytes. **(E)** PKA activity in primary cultured cardiomyocytes under a hypoxic condition. Data are presented as mean ± SEM, ^#^*P* < 0.05 *vs* sham, *n* = 6; ^*^P < 0.05 *vs* MI, *n* = 8. *P* values were analyzed using one-way ANOVA.

Drp1 is a critical regulator of mitochondrial fission, which typically resides in an inactive form in the cytosol, and when phosphorylated at Ser616, it can be activated and translocated into mitochondria. To confirm the changes of mitochondrial morphology observed in our models indeed representing the mitochondrial fission, we measured Drp1 activities by determination of the protein level of Ser616-phosphorylated form of Drp1 (p-Drp1^Ser616^) in cardiomyocytes, which has been commonly used as an indication of mitochondrial fission [26, 27]. Our western blot analysis showed no significant change in the total protein level of Drp1 (t-Drp1) in MI hearts (Figure 2A), but a significant increase in p-Drp1^Ser616^ protein level in MI compared to sham-operated hearts. Notably, administration of Apelin diminished such an MI-induced increase in p-Drp1^Ser616^ protein level without affecting t-Drp1 (*P* < 0.01; Figure 2A & 2B). Consistent with the above *in vivo* results, the expression of p-Drp1^ser616^ was also markedly increased in primary cardiomyocytes cultured under a hypoxic condition to mimic the major insult of ischemia at the cellular level and abrogated by Apelin treatment (Figure 2C & 2D), while the expression of t-Drp1 remained unaltered.

It has been reported that PKA stimulates Drp1 phosphorylation and induces mitochondrial fission in hypoxia [28]. We therefore tested the effect of Apelin on PKA activity. As depicted in Figure 2E, Apelin inhibited the PKA activity in primary cultured cardiomyocytes under a hypoxic condition.

### Apelin attenuates apoptosis of cardiomyocytes through suppressing the mitochondrial death pathway

Increasing evidence indicates that mitochondrial fission plays a crucial role in inducing apoptosis [27]. Inhibition of mitochondrial fission by Apelin is therefore expected to produce anti-apoptotic effects. To test this hypothesis, we carried out the following experiments.

**Figure 3:**
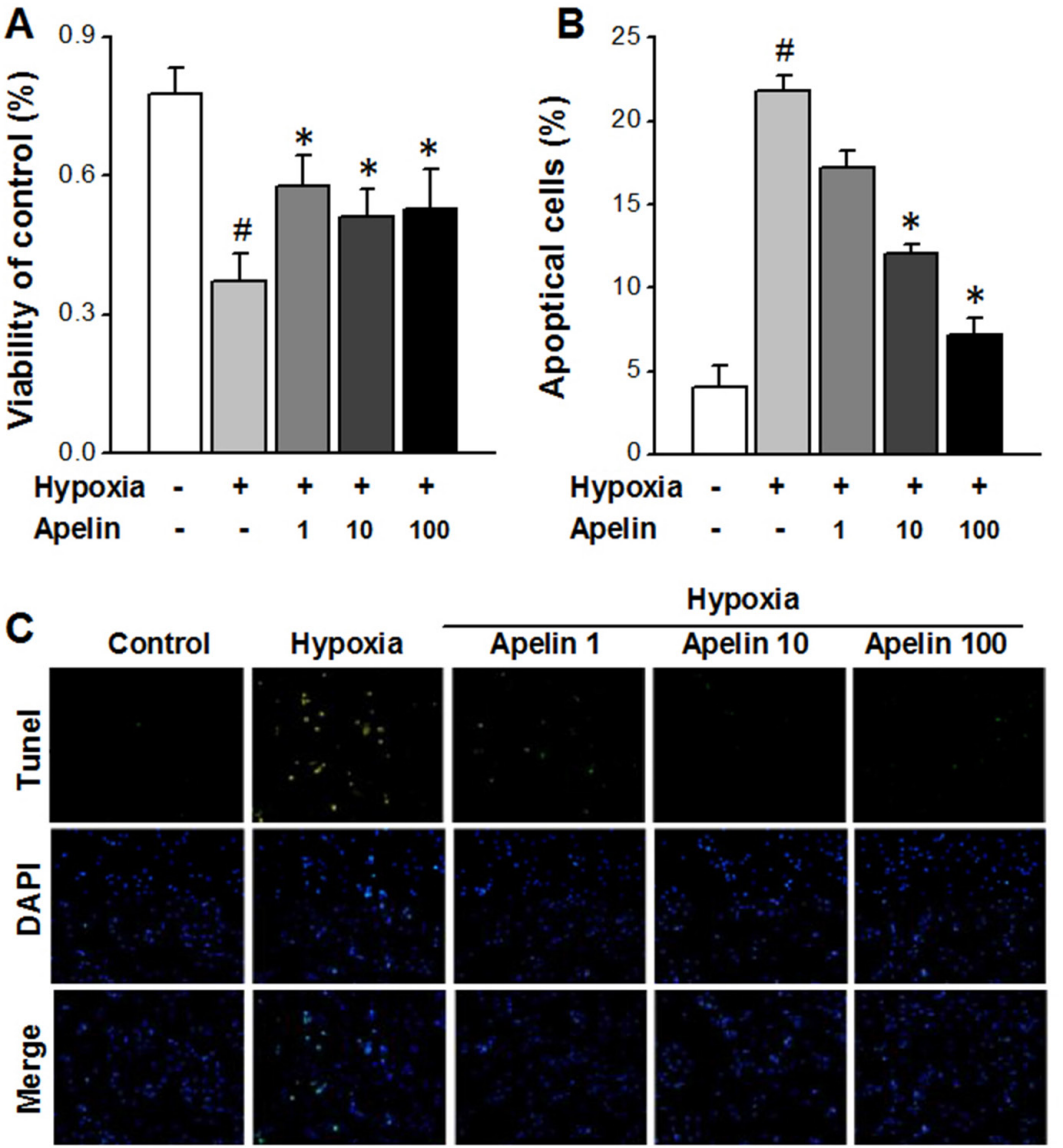
Apelin attenuates apoptosis of cardiomyocytes through suppressing the mitochondrial death pathway **(A)** The viability of cells in each groups. **(B)** TUNEL photomicrographs of cardiomyocyte in different groups (magnification ×100). **(C)** Percentage of TUNEL-positive cells in each group. Data are presented as mean ± SEM. ^#^*P* < 0.05 *vs* sham, *n* = 6; ^*^P < 0.05 *vs* MI, *n* = 6. *P* values were analyzed using one-way ANOVA.

First, we investigated the effects of varying concentrations of Apelin on cell viability by MTT assay. The results shown that hypoxia decreased cardiomyocyte viability (Figure 3A), and Apelin rescued this damaging effect in a concentration-dependent manner.

Second, in order to clarify if the changes of cell viability were attributable to apoptotic cell death, we used TUNEL staining to identify chromosomal fragmentation and quantify apoptosis. As depicted in Figure 3B, hypoxia caused a marked increase in the number of TUNEL positive cells, indicating the induction of apoptosis, and such an increase was mitigated following treatment with Apelin (*P* < 0.05 Apelin/hypoxia *vs* hypoxia).

**Figure 4:**
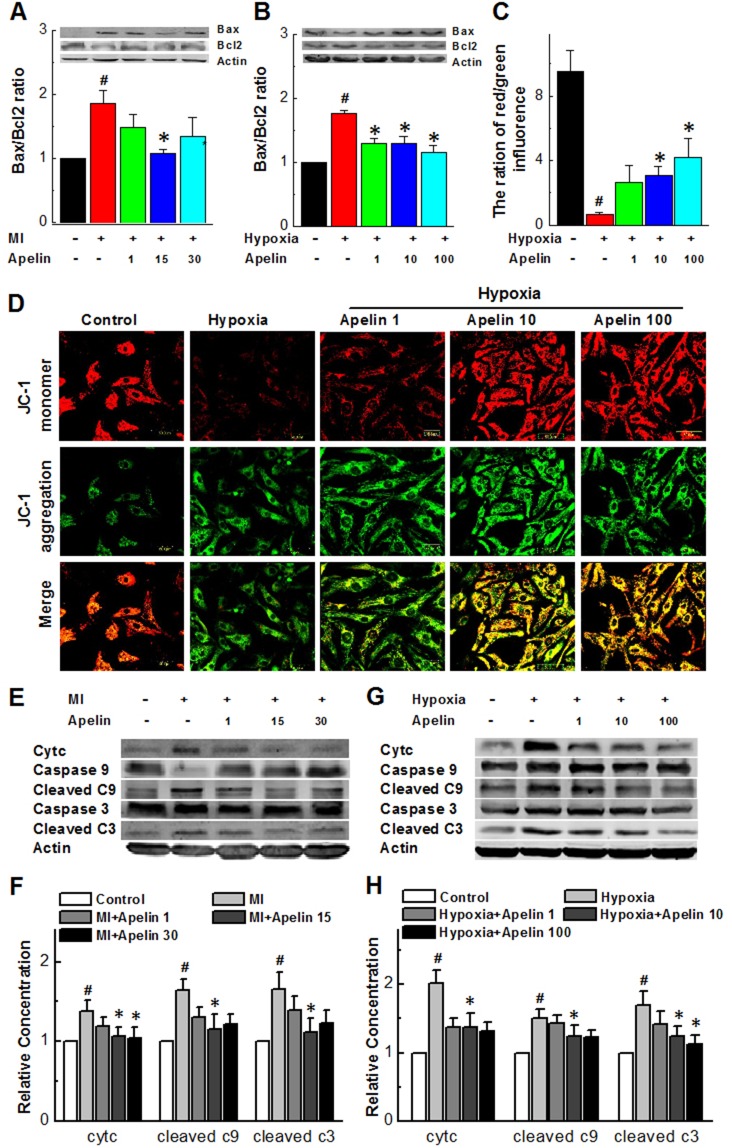
Apelin attenuates apoptosis of cardiomyocytes through suppressing the mitochondrial death pathway **(A)** Representative Western blot band showing levels of Bax and Bcl-2 in MI mice **(B)** Representative Western blot band showing levels of Bax and Bcl-2 in cultured neonatal cardiomyocytes. **(C)** The ratio of red and green influence in the change of mitochondrial membrane high potential (red) and low potential (green). **(D)** Representative fluorescent images of JC-1 under a hypoxic condition. **(E** and **F)** Representative Western blot band showing levels of caspase3, cleaved caspase-3, cytochrome C, caspase9, cleaved caspase-9 in the areas proximal to infarct/ischemic zone in MI mice. **(G** and **H**) Quantification of cleaved caspase-3, cytochrome C, cleaved caspase-9 in the areas proximal to infarct/ischemic zone in cultured neonatal mice cardiomyocytes. Data are presented as mean ± SEM. ^#^*P* < 0.05 *vs* sham, *n* = 6; ^*^P < 0.05 *vs* MI, *n* = 6. *P* values were analyzed using one-way ANOVA.

Next, we sought to determine whether the mitochondrial apoptosis pathway was involved in the MI injury in our models. To this end, we determined the changes of expression of two mitochondria-associated signaling molecules, a pro-apoptotic protein Bax and an anti-apoptotic protein Bcl-2 in MI hearts with and without Apelin. While the Bax/Bcl-2 ratio significantly increased in MI mice and attenuated by Apelin treatment (Figure 4A). Qualitatively the same results were consistently observed under *in vitro* conditions: Bax/Bcl-2 ratio was significantly increased in hypoxia and reversed in the presence of Apelin (Figure 4B).

The fate of cells succumbing to the intrinsic pathway of apoptosis is sealed by mitochondrial membrane potential (MMP) and loss of MMP is an important event in the mitochondrial apoptosis pathway [29]. As shown in Figure 4C-4D with the results of JC-1 staining to reflect the MMP status, hypoxia caused a robust decrease in red fluorescence signal, suggesting a loss of MMP in cultured cardiomyocytes and such deleterious change was pronouncedly diminished in a concentration-dependent manner when treated with Apelin for 12 h, indicating the protective effect of Apelin on the integrity of mitochondria.

It is known that rupture of mitochondrial membrane or loss of MMP can eventually result in release of cytochrome C from the inner membrane space of mitochondria to the cytosol and subsequent activation of the caspases-9 that ultimately leads to activation of caspase-3, an executioner of apoptosis [30]. Our data depicted in Figure 4E-4H indeed provide the evidence for such a process in cardiomyocytes exposed to a hypoxic condition: hypoxia increased release of cytochrome C and the levels of cleaved or activated caspase 9 and caspase 3 (*P* < 0.05 *vs* sham). These damaging alterations were reversed by Apelin (*P* < 0.05 *vs* MI).

### Apelin improves cardiac function in MI mice

The results presented above demonstrate that p-Drp1^ser616^-mediated mitochondrial fission is enhanced in MI mice, and inhibition of p-Drp^ser616^ by Apelin reduces mitochondrial fission and consequently reduces cardiomyocyte apoptosis. These findings suggest that Apelin possesses a cardioprotective effect against MI injury and such a beneficial action could manifest at the level of whole heart and overall cardiac function. To test this notion, we conducted the following experiments.

**Figure 5:**
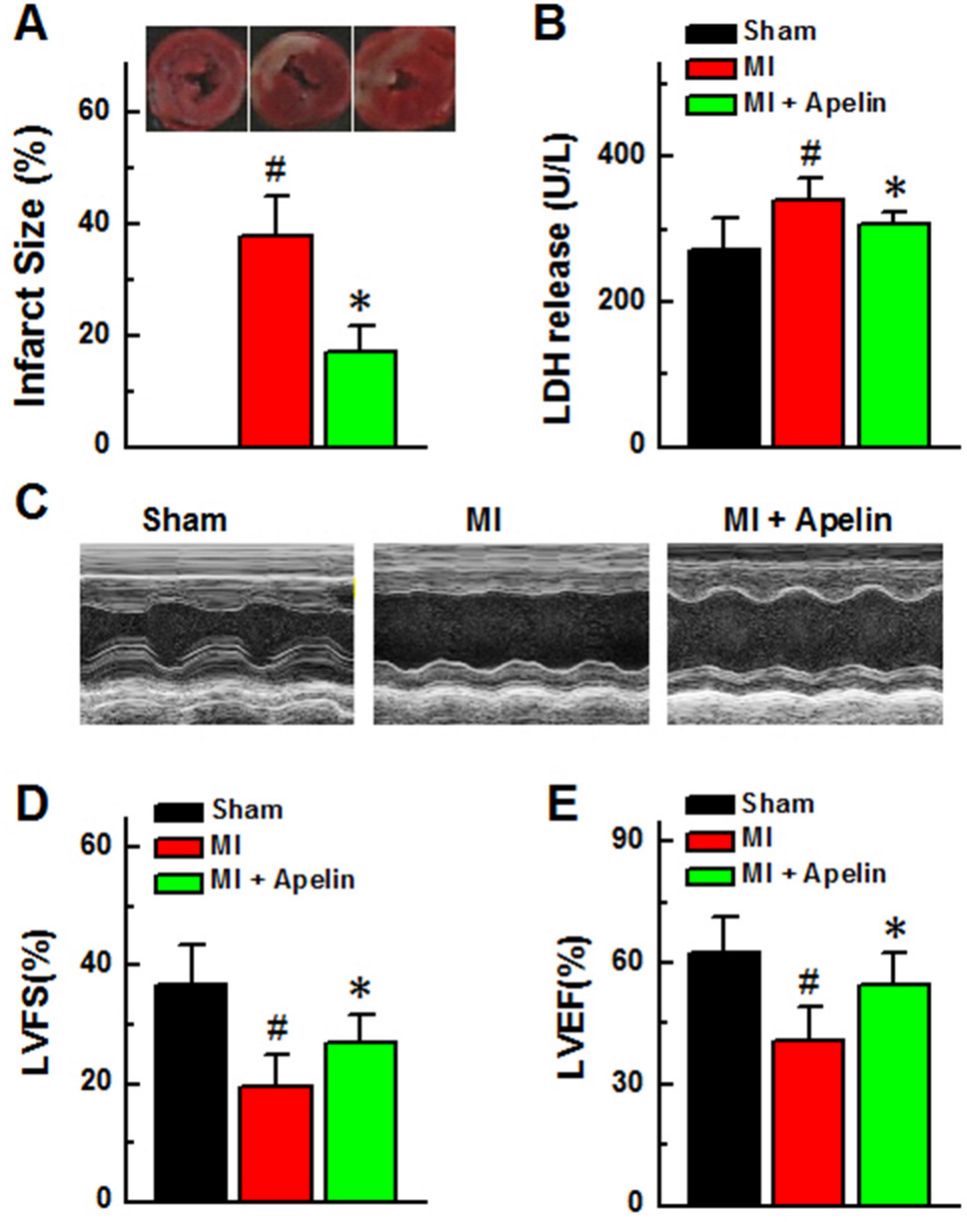
Apelin improves cardiac function in MI mice **(A)** Myocardial infarct sizes in each group and quantification of infarct sizes. Scale bar, 2mm. **(B)** LDH release. **(C)** Representative echocardiographic image. **(D)** Left ventricular fractional shortening. **(E)** Left ventricular ejection fraction. Data are presented as mean ± SEM. ^#^*P* < 0.05 *vs* sham, *n* = 6; ^*^P < 0.05 *vs* MI, *n* = 6. *P* values were analyzed using one-way ANOVA.

First, we measured the changes of infarct size with or without Apelin. As demonstrated in Figure 5A, MI mice treated with Apelin showed a significant decrease in infarct size compared with non-treated MI mice (16.9 ± 4.75 *vs* 37.9 ± 10.1%, *n* = 8, *P* < 0.05).

Second, we detected changes of serum LDH levels in MI mice with or without Apelin, as an index of overall cardiac damage. The MI mice received Apelin had considerably lower LDH levels compared with those without Apelin treatment (306 ± 27 *vs* 341 ± 29 U/L, *P* < 0.05; Figure 5B).

Finally, we investigated the effects of Apelin on cardiac dysfunction induced by MI using echocardiography. As shown in Figure 5C-5E, administration of Apelin restored the MI-induced decreases in EF and FS (EF: 54.7 ± 7.6% *vs* 40.9 ± 8.1%; FS: 26.8 ± 5.0 *vs* 19.5 ± 2.7, *P* < 0.05).

## DISCUSSION

In the present study, we found that Apelin reduced ischemia-induced cardiac injury and improved cardiac function via inhibiting Drp1-mediated mitochondrial fission and its downstream apoptotic signaling, strongly suggesting that the inhibition of Drp1 would be a novel endogenous anti-apoptotic mechanism and promising cardiac protective strategy by which preserves the integrity of mitochondrial membrane and inhibits mitochondria-mediated apoptosis.

Mitochondria are dynamic organelles that undergo cycles of fusion and fission to maintain their morphology and function. Drp1 is one of the proteins proposed to participate in the fission process. Regulation of expression and activity of Drp1 affects mitochondrial fission and mitochondria-dependent apoptosis [31]. Drp1 presents mainly in an unassembled form in the cytosol, but in response to different stimuli such as cardiac ischemia, it can be recruited to the mitochondrial outer membrane and induces mitochondrial fragmentation prior to caspase activation by the release of cytochrome C [32]. It has also suggested that regulation of Drp1 by phosphorylation is important for Drp1 cycling between the cytosol and mitochondria [32]. Two major phosphorylation sites were found in Drp1. When Drp1 was phosphorylated at Ser616 residue, mitochondrial fission occurred, whereas phosphorylation at Ser637 blocked fission and induced fusion and elongation of mitochondria. Drp1 was indicated as one of the PKC-δ substrates and interacting proteins. Recent studies implicated the phosphorylation of Drp1 by CDK5 and PKA promote astrocytes apotosis in the rat hippocampus. Taguchi et al. reported that human Drp1 can be activated by Cdk1/cyclin B-mediated phosphorylation at the site of Ser616 in the variable domain leading to Drp1-dependent mitochondrial fission [33]. In the present study, we found that the level of p-Drp1^Ser616^ increased after ischemia and under condition of hypoxia accompanied by mitochondrial fission and these alternations could be mitigated dramatically in the presence of Apelin. Moreover, Apelin effectively prevented cardiomyocyte apoptosis by inhibiting the release of cytochrome C and subsequent activation of the caspase-9 - caspase-3 cascade. Furthermore, Apelin markedly improved cardiac function as the result of infarct size as well as serum LDH level reductions in MI mice. To the best of our knowledge, this study represents the first evidence for the cytoprotective action of Apelin in ischemic injury and inhibition of Drp1 as a mechanism underlying its anti-apoptotic property. Our study extends the findings by Ong *et al* and suggests that inhibition of Drp1 has a therapeutic potential for patients diagnosed with ischemic heart disease.

Several studies about apoptosis revealed the causal relationship between mitochondrial fission and the induction of apoptosis. In our study, we observed that hypoxia induced mitochondrial fission and cardiomyocyte apoptosis, which could be suppressed by Apelin through either downregulating p-Drp1^Ser616^ level or suppressing Drp-1 activation, or both. This effect resulted in the inhibition of the cytochrome C release and subsequent activation of the caspase-9 - caspase-3 cascade. These results showed that Apelin improved mitochondrial dysfunction and attenuated mitochondria-dependent apoptosis. Likely by acting on this signaling pathway, Apelin reduced infarct size and improved the impaired cardiac function induced by ischemia. This is in agreement with the notion from Rastaldo *et al.* that Apelin could limit infarct size and improve cardiac post-ischemic mechanical recovery only if given after ischemia [24].

In addition to the mitochondrial death pathway, Apelin has also been documented to act via other singling mechanisms. For instance, Tao *et al.* reported that Apelin protects the heart against I/R injury through inhibition of the ER (endoplasmic reticulum)-dependent apoptotic pathways [25]. In such a circumstance, Apelin might inhibit the opening of mitochondrial permeability transition pore and stimulate the reperfusion injury RISK pathway by inactivating GSK-3β to activate PI3K/Akt and ERK1/2. It is possible that such a pathway is also involved in our models, and this requires separate studies to verify.

In summary, our data indicated that Apelin decreased p-Drp1^Ser616^ level likely by suppressing calcineurin activity, leading to inhibition of mitochondria fission. This beneficial effect may play a key role in maintenance of the integrity of mitochondrial membrane and further to protect from the loss of MMP so as to inhibit the mitochondria-mediated apoptosis. Future investigation should be undertaken to address the detailed molecular mechanisms by which Apelin interacts with Drp1 and links between Drp1 and the mitochondrial death pathway.

## MATERIALS AND METHODS

### Animals

All animal experiments were approved by the Committee on Animal Experimentation of Harbin Medical University (Approval ID: DEC6121) and were conducted in accordance with the international guidelines regarding animal experimentation. Male C57BL6/J mice aged 8-10 weeks and weighing 20-25 g were obtained from the Animal Centre of Harbin Medical University. The mice were maintained in a pathogen-free, temperature-controlled animal house with a 12:12-h light/dark cycle, and given free access to a standard diet and tap water. Mice were randomly divided into four groups: sham-operated rats (control group, *n* = 8), myocardial infarction group (MI group, *n* = 8), and MI with Apelin group (MI + Apelin groups (1, 15, and 30 μg/kg, respectively; *n* = 8).

### Chemicals

Apelin was obtained from Sigma Company (A6469, Sigma, china), Apelin stock solution was prepared in double polished distilled water (ddH_2_O) and to prepared relevant concentration.

### Mouse model of myocardial infarction

Adult male C57BL/6J mice were treated with Apelin for 4 weeks (*i.p.* 1, 15, 30 μg/kg/day, respectively) and then subjected coronary artery ligation (LAD) to induce MI. MI model was carried out by LAD as described elsewhere [34]. In brief, mice were anaesthetized with a mixture of xylazine (5 mg/kg, i.p) and ketamine (100 mg/kg, i.p) and the depth of anesthesia was assessed by monitoring the pedal withdrawal reflex. Mice were then ventilated and subjected to a left-sided thoracotomy and the left coronary artery ligation to induce MI. Sham-operated rats underwent an identical surgical procedure without LAD ligation. After 6 hours of LAD ligation, we evaluate their cardiac function and measure the infarct size using hearts tissue. The heart was quickly excised and weighted in cold (4°C) buffer. The left ventricle tissue was then rapidly frozen in liquid nitrogen and stored at -80°C for subsequent western blot analysis.

### Echocardiographic measurements of cardiac function

After 6 h ligation of LAD, mice were anaesthetized with sodium pentobarbital (50 mg/kg, i.p, Merck). We used transthoracic echocardiography with an ultrasound machine (Vivid 7, GE Medical, Horten, Norway) equipped with a 10-MHz phased-array transducer to test left ventricular ejection fraction (EF), and fractional shortening (FS) were calculated from M-mode recording.

### Measurement of infarct size by tetrazolium chloride staining

Myocardial infarct size was evaluated by 2, 3, 5-triphenyltetrazolium chloride triazole (TTC) (Solarbio, Beijing, China) staining. Briefly, after 6 h ligation, mice were euthanized and hearts were removed rapidly to measure infarct size and transmission electron microscopy. After wash with phosphate buffer solution (PBS, pH 7.4) and frozen at -20°C for 2 h, the left ventricular (LV) wall was cut into 6 pieces with cross-section of 2-mm thickness. The preparations were incubated in 1% TTC in 0.1 mol/L PBS at 37°C for 15 min, and subsequently fixed in 10% formalin solution (pH 7.4) for 12 h. The infarct area (IS, white color) and area at risk (AAR, red color) were identified.

### Transmission electron microscopic study

Mice were evaluated cardiac function after LAD, hearts were cut into pieces and fixed in 0.1 mol/L sodium phosphate buffer containing 2.5% glutaraldehyde for 3 h at 4°C, then osmicated in 1% osmium tetroxide for 1 h at 4°C. After dehydration of the tissues by using ethanol series, the samples were embedded in Epon 812 and sectioned using a Leica EM UC6 (Leica Coviema, Austria) ultra-microtome. Sections were viewed and photographed using a Hitachi 7650 TEM (Hitachi, Tokyo, Japan) at 80 kv.

### Western blot analysis

Western blotting was performed as previously described [35]. Briefly, cells were lysed with modified RIPA buffer on ice for 30 min, and cell lysates were subsequently centrifuged at 12,000 g at 4°C for 20 min. The supernatant was transferred to a fresh ice-cold tube, and protein concentrations were determined via a Bio-Rad protein assay. Equal concentrations of protein samples were mixed with SDS sample buffer and denatured at 95°C for 5 min. Samples were resolved in 8% SDS-page gel. Gels were transferred onto nitrocellulose membranes that were then blocked with 5% non-fat dried milk in TTBS (0.1% Tween-20) for 1 h. Following blocking, the blots were incubated with primary antibodies at 4°C overnight. The antibodies against Bax, Bcl-2, total Drp1 protein (t-Drp1; Proteintech Group, Chicago, USA), phosphorylated form of Drp1 at Ser616 (p-Drp1^ser616^), cytochrome C, caspase 9, and caspase 3, were obtained from Santa Cruz Biotech (Santa Cruz, CA, USA). Following washing with TTBS, the membranes were incubated with HRP-conjugated secondary antibodies (1:5,000, Santa Cruz Biotechnology) for 1 h. Finally, the membranes were washed again with TTBS, and the blots were determined using an enhanced chemiluminescence detection system (ECL; GE Healthcare, Bio-Science AB, Uppsala, Sweden).

### Cell culture

Hearts from 2∼3-day old mice were finely minced and digested using type II collagenase (120 units/ml; Worthington Biochemical Corp., Lakewood, NJ) for 10 min, and supernatant was collected. Cardiomyocytes were maintained in DMEM supplemented with penicillin and streptomycin (1%) and fetal bovine serum (FBS; 10%). Cardiomyocytes at the 2nd passage were used in our experiments.

Primary cultures of neonatal mice cardiomyocytes were prepared from 1∼3-day-old Wistar rats by trypsin. Briefly, the ventricular myocardium was minced in DMEM (Hyclone, City, USA). After each of six successive incubations, the cells were suspended in DMEM containing 10% FBS (Hyclone) and centrifuged. Pooled cells were plated onto dishes at a density of 1×10^5^ cells/cm^2^ and incubated at 37°C in humidified air with 5% CO_2_ for 72 h, and 0.1 mM bromodeoxyuridine (Sigma, St. Louis, USA) was added into the medium to deplete non-cardiomyocytes. Before experiments, the cells were starved for 24 h and then treated with Apelin at varying concentrations (1, 10 and 100 nM) for 12 h. Then induced by hypoxia for 12h, cell viability was determined by MTT assay.

### Measurement of serum LDH levels

Cytotoxicity was measured by lactate dehydrogenase (LDH) release into the blood serum using a commercially available kit (Jiancheng Bioengineering Institute, Nanjing, China), and assessed according to the following equation: LDH release = (LDH activity in the medium/total LDH activity) × 100%.

### Measurement of the mitochondrial membrane potential (MMP)

The lipophilic cationic probe JC-1 (Beyotime, Shanghai, China) was employed to measure the mitochondrial membrane potential (ΔΨm) of primary mice cardiomyocytes according to the manufacturer’s directions. Briefly, cells were incubated with JC-1 staining solution (5 μg/mL) at 37°C for 20 min and rinsed twice with JC-1 staining buffer. Then the fluorescence intensity of both mitochondrial JC-1 monomers (excitation wavelength λex 514 nm and emission wavelength λem 529 nm) and aggregates (λex 585 nm and λem 590 nm) was detected using a monochromatic microplate reader (Safire II, Tecan, City, Switzerland). ΔΨm was calculated as the ratio of red to green fluorescence.

### TUNEL staining

Cell apoptosis was detected with TUNEL assay (*In Situ* Cell Death Detection Kit, Roche, Indianapolis, Switzerland). DNA fragmentation of individual cells was further detected *in situ* by TUNEL. Cells were observed by a fluorescence microscope (Eclipse 80I, Nikon, City, Japan).

### Statistical analysis

All values are expressed as the mean ± SEM. Differences between two groups were determined via a Student’s *t*-test. Comparisons between more than two groups, data were assessed using one-way ANOVA followed by a Bonferroni’s correction. A value of *P* < 0.05 was considered as statistically significant.

## Abbreviations

(Drp1): dynamin-related protein 1
(MI): myocardial infarction
(LAD): coronary artery ligation
(p-Drp1^ser 616^): phosphorylated Drp1
(MMP): mitochondrial membrane potential
(APJ): apelin related receptor
(RISK): salvage kinase
